# The pro-domains of neurotrophins, including BDNF, are linked to Alzheimer's disease through a toxic synergy with Aβ

**DOI:** 10.1093/hmg/ddv130

**Published:** 2015-05-07

**Authors:** Jung Yeon Lim, Charles P. Reighard, Damian C. Crowther

**Affiliations:** 1 Department of Genetics, University of Cambridge, Downing Street, CambridgeCB2 3EH, UK,; 2 Department of Biochemistry, University of Cambridge, Tennis Court Road, CambridgeCB2 1GA, UK and; 3 MedImmune Limited, Aaron Klug Building, Granta Park, CambridgeCB21 6GH, UK

## Abstract

Brain-derived neurotrophic factor (BDNF) has a crucial role in learning and memory by promoting neuronal survival and modulating synaptic connectivity. BDNF levels are lower in the brains of individuals with Alzheimer's disease (AD), suggesting a pathogenic involvement. The *Drosophila* orthologue of BDNF is the highly conserved Neurotrophin 1 (DNT1). BDNF and DNT1 have the same overall protein structure and can be cleaved, resulting in the conversion of a full-length polypeptide into separate pro- and mature-domains. While the BDNF mature-domain is neuroprotective, the role of the pro-domain is less clear. In flies and mammalian cells, we have identified a synergistic toxic interaction between the amyloid-β peptide (Aβ_1–42_) and the pro-domains of both DNT1 and BDNF. Specifically, we show that DNT1 pro-domain acquires a neurotoxic activity in the presence of Aβ_1–42_. In contrast, DNT1 mature-domain is protective against Aβ_1–42_ toxicity. Likewise, in SH-SY5Y cell culture, BDNF pro-domain is toxic only in the presence of Aβ_1–42_. Western blots indicate that this synergistic interaction likely results from the Aβ_1–42_-induced upregulation of the BDNF pro-domain receptor p75^NTR^. The clinical relevance of these findings is underlined by a greater than thirty fold increase in the ratio of BDNF pro- to mature-domains in the brains of individuals with AD. This unbalanced BDNF pro:mature-domain ratio in patients represents a possible biomarker of AD and may offer a target for therapeutic intervention.

## Introduction

Alzheimer disease (AD) is a devastating neurodegenerative disorder that manifests as a progressive decline in cognitive function (1). Pathologically, AD is characterized by the accumulation of plaques containing amyloid-β (Aβ), tau-laden neurofibrillary tangles and progressive synaptic and neuronal loss. Emerging evidence suggests that brain-derived neurotrophic factor (BDNF) may be involved in the pathogenesis of AD. BDNF is the most functionally diverse member of the neurotrophin (NT) family. As well as promoting the differentiation and migration of peripheral and central neurons and glia during development (2,3), BDNF also supports neuronal survival and synaptic plasticity in adulthood and also having effects on memory and mood (4,5).

All NTs are synthesized as precursors (pro-NTs) that then form homodimers (6). Pro-NTs may be secreted from cells (7) or cleaved intracellularly by furin or proconvertases to yield the mature C-terminal NT. The processing of pro-BDNF may also be completed extracellularly by plasmin or metalloproteinases, including MMP3, MMP7 and MMP9 (7,8). BDNF exerts many of its neuroprotective effects by binding to the TrkB receptor, a receptor tyrosine kinase (9). However, the consequences of its binding to its alternative receptor p75^NTR^ are thought to include promotion of myelination (3) and neuronal migration (10) but also neuronal process retraction (11) and neuronal apoptosis (12). While the application of mature BDNF reduces sensory neuronal loss in dorsal root ganglia after peripheral nerve injury, in contrast the precursor form of BDNF increases the death of axotomized neurons (13). This toxic effect requires the expression levels of the TrkB and p75^NTR^ high-affinity receptors. Likewise, the pro- and mature-domains of nerve growth factor (NGF) have different effects on neurones, specifically the precursor form of NGF preferentially activates p75^NTR^ resulting in apoptosis, while the NGF mature-domain signals through the TrkA receptor resulting in neurotrophic activity (7).

Reduced levels of hippocampal and cortical pro-BDNF or BDNF are a consistent feature of AD (14,15); however, the mechanistic basis for this correlation is unclear. There is some evidence that that Aβ may reduce TrkB-mediated neurotrophic signaling (16); conversely, a reduction in BDNF may favor the amyloidogenic processing of the amyloid precursor protein, favouring Aβ generation (17). However, little is known regarding the particular role of BDNF pro-domain despite it harbouring a common sequence polymorphism (Val66Met) linked to accelerated cognitive decline in AD (18,19). DNT1 is the *Drosophila* orthologue of BDNF and as a member of the NT superfamily it has structural and sequence orthologues throughout the phylogenetic spectrum (20).

In the present study, we investigate a potential role for the DNT1/BDNF pro-domain in mediating Aβ toxicity and, in clinical samples, show that AD is accompanied by profound changes in the pro:mature-domain ratio of BDNF.

## Results

### Drosophila DNT1 mRNA levels are high during development but suppressed in adulthood

**Figure 1.**
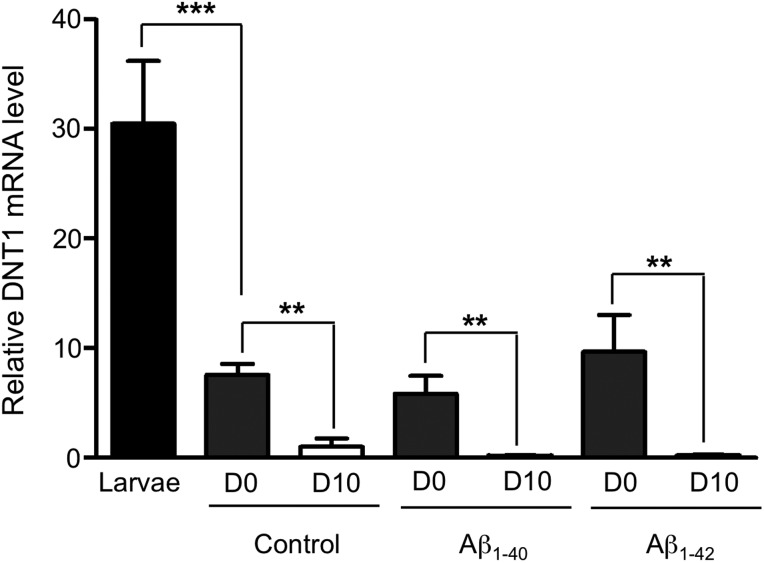
*Drosophila* DNT1 mRNA levels are high during development but suppressed in adulthood. (**A**) DNT1 and RP49 mRNA were measured at Days 0 (D0) and 10 (D10) by quantitative real-time PCR in control flies and those expressing the Aβ_1–40_ and Aβ_1–42_ peptides. DNT1 mRNA levels were highest during larval development (black bar) and fall rapidly before eclosion (Day 0, grey bars). An essentially identical pattern of age-related decline in DNT1 was seen in ‘Control’ flies (elav-gal4; +) and in ‘Aβ_1-40_’ (elav-gal4; UAS-Abeta40) and ‘Aβ_1–42_’ (elav-gal4; UAS-Abeta42) flies. Data are presented as the ratio of DNT1 to RP49 mRNA levels (±SD). Statistical comparisons were made by one-way analysis variance test. **P < 0.01 and ***P < 0.001.

Low levels of BDNF have been correlated with increased age-related risk of AD (16,21) and so we used quantitative RT-PCR to quantify the levels of DNT1 mRNA in flies during development and for the first 10 days of adult life. We found that levels of endogenous *DNT1* mRNA were high during development but fell rapidly in adult life whether or not Aβ was also co-expressed (Fig. 1). Consequently knock-down of DNT1, by RNAi or the hemizygous introduction of a null allele (Supplementary Material, Fig. S1A), had no effect on longevity and locomotor phenotypes in the adult (Supplementary Material, Fig. S1B–E).

### The subdomains of DNT1 have differential effects on neurotoxic phenotypes in the fly

**Figure 2.**
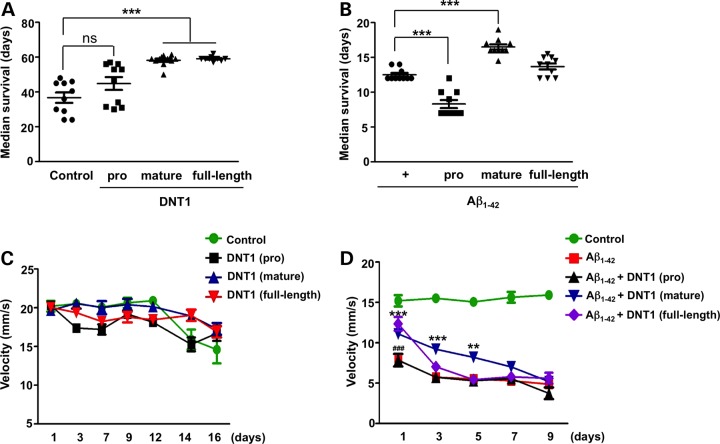
The subdomains of DNT1 have differential effects on neurotoxic phenotypes in the fly. (**A**) Expression of full length (elav-gal4; UAS-DNT1 full length; +) and mature-domain (elav-gal4; UAS-DNT1 mature-domain; +) of DNT1 prolonged the lifespan of control flies (elav-gal4; +; +), whereas DNT1 pro-domain (elav-gal4; UAS-DNT1 pro-domain; +) had no effect. The flies were cultured at 29°C. Each graph presents the mean of 10 estimates of median survival ±SEM. Statistical comparisons were made by one-way analysis variance test. ***P < 0.001. (**B**) The overexpression of DNT1 mature-domain (elav-gal4; UAS-DNT1 mature-domain; UAS-ArcAbeta42) significantly increased median survival of Arc Aβ_1–42_ flies (elav-gal4; +; UAS-ArcAbeta42) while DNT1 pro-domain (elav-gal4; UAS-DNT1 pro-domain; UAS-ArcAbeta42) further reduced median survival by 30%. Full-length DNT1 (elav-gal4; UAS-DNT1 full length; UAS-ArcAbeta42) had no overall effect. The flies were cultured at 29°C. Each graph presents the mean of 10 estimates of median survival ±SEM. Statistical comparisons were made by one-way analysis variance test. ***P < 0.001. (**C**) The mean climbing velocity of control flies was not affected by the overexpression of any DNT1 constructs. The genotypes were the same as (B). Mean velocity ±SEM. (**D**) Some rescue of the locomotor deficits associated with Arc Aβ_1–42_ expression (red squares) was seen upon co-expression of full-length DNT1 (purple diamonds, ^###^*P* < 0.001 on Day 1 only) and DNT1 mature-domain (blue inverted triangles, **P < 0.01 and ***P < 0.001 on days 1, 3 and 5), but not DNT1 pro-domain (black triangles). The genotypes were the same as (B). Mean climbing velocities were lower in all Arc Aβ_1–42_ flies when compared with controls at all times points. Mean velocity ±SEM. Statistical comparisons were made by two-way analysis variance test. **P < 0.01 and ***P < 0.001.

Exogenous application of BDNF improves cognitive and behavioural deficits and ameliorates Aβ pathology in murine and primate models of AD (22,23). To test whether overexpression of DNT1 rescues neurotoxic phenotypes induced by Arc Aβ_1–42_ peptide in flies, we expressed three forms of the protein (20): (1) full-length DNT1, (2) pro-domain of DNT1, (3) mature-domain of DNT1 in all neurons and measured the interaction with Arc Aβ_1–42_ expression. The overexpression of both mature- and full-length DNT1 prolonged the lifespan of control flies at 29°C, increasing their median survival by 30%. Notably, overexpression of DNT1 pro-domain had no effect on the longevity of control flies (Fig. 2A). As expected Arc Aβ_1–42_-expression resulted in a 50% reduction in longevity and this effect was significantly suppressed by co-expression of DNT1 mature-domain (Fig. 2B). Remarkably, the co-expression of DNT1 pro-domain with Arc Aβ_1–42_ enhanced the reduction in longevity, significantly reducing the median survival by a further 30%. Interestingly when full-length DNT1, containing both pro- and mature-domains, was co-expressed with Arc Aβ_1–42_, the effects of the component domains cancelled each other out, resulting in no overall effect on the longevity (Fig. 2B). These results were confirmed at 25°C when transgene expression levels were expected to be lower (Supplementary Material, Fig. S2).

To further investigate the effect of DNT1 on Aβ-linked toxicity, we assessed the average climbing velocity of flies overexpressing three forms of DNT1 in the absence, or presence, of the Arc Aβ_1–42_. Wild-type control flies showed the expected age-related reduction in climbing velocity that is not modified by the overexpression of any DNT1 construct (Fig. 2C). Arc Aβ_1–42_ flies exhibited a much earlier and more rapid decline in locomotor activity but this was not affected by the overexpression of DNT1 pro-domain (Fig. 2D). However, full-length DNT1 and the mature-domain of DNT1 did rescue locomotor deficits as witnessed by higher velocities from Days 1–5 when compared with flies expressing Arc Aβ_1–42_ alone. No activity assay could be performed for Arc Aβ_1–42_ flies beyond Day 9 due to their short life span.

### Co-expression of various domains of DNT1 does not change the abundance or conformation of Aβ

**Figure 3.**
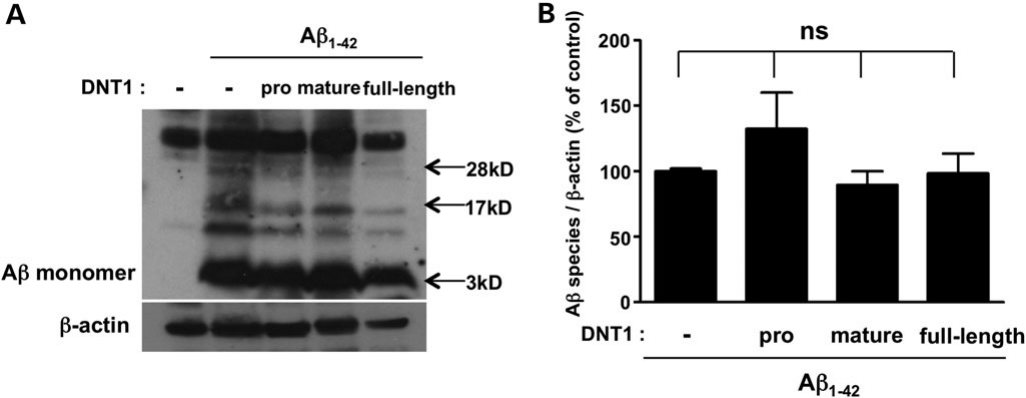
Co-expression of various domains of DNT1 does not change the abundance or conformation of Aβ. (**A**) Western blots of SDS–PAGE gels of *Drosophila* brain extracts containing the SDS-soluble Aβ fraction. Immunodetection using the monoclonal antibody 6E10 revealed a number of bands in all Arc Aβ_1–42_ lines. There were no consistent changes in the individual Aβ bands between genotypes across three independent experiments. β-Actin expression was used as a loading control. (**B**) The multiple Aβ-specific band intensities were normalized to β-actin and the data were plotted as a bar chart (band intensities ±SEM, *n* = 3). There was no consistent change in the total intensity of Aβ bands upon co-expression of various domains of DNT1. Genotypes: ‘−’: elav-gal4; +; UAS-Abeta42, ‘pro’: elav-gal4; UAS-DNT1 pro-domain; UAS-Abeta42, ‘mature’: elav-gal4; UAS-DNT1 mature-domain; UAS-Abeta42 and ‘full length’: elav-gal4; UAS-DNT1 full length; UAS-Abeta42.

To understand whether the modulation of Aβ toxicity results from changes in the handling of the peptide, we western blotted SDS–PAGE gels of brain homogenates using the 6E10 monoclonal antibody (Fig. 3A, representative of *n* = 3). While multiple Aβ-specific bands representing monomeric, dimeric and oligomeric forms were visible in all Arc Aβ_1–42_ lines, there was no consistent change in their intensities, relative to each other or to the β-actin control, upon expression of the various domains of DNT1 (Fig. 3B). Thus, it appeared unlikely that DNT1 constructs were regulating the clearance or aggregation of Aβ in the fly brain and so alternative mechanisms were investigated in mammalian cell culture.

### The pro-domain of BDNF and Aβ_1–42_ exhibit synergistic toxicity in SH-SY5Y human neuroblastoma cell cultures

**Figure 4.**
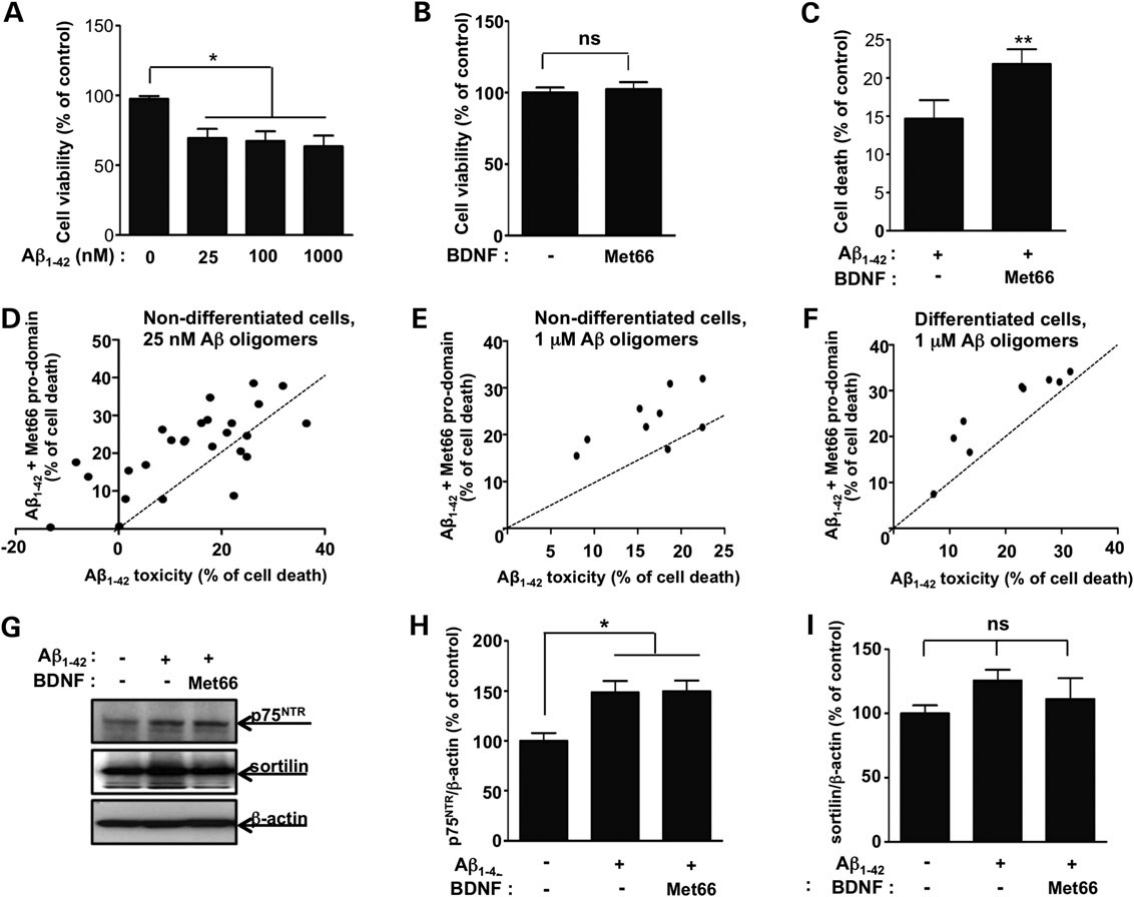
The pro-domain of BDNF and Aβ_1–42_ exhibits synergistic toxicity in SH-SY5Y human neuroblastoma cell cultures. (**A**) Addition of 25, 100 and 1000 nm oligomeric Aβ_1–42_ to the culture medium of SH-SY5Y human neuroblastoma cells for 48–60 h resulted in significant toxicity when compared with vehicle. Each bar represents mean ± SEM (*n* = 3). Statistical comparisons were made by one-way analysis variance test. * *P* < 0.05. (**B**) Cells were treated with 200 ng/ml recombinant pro-domain of BDNF for 48–60 h. Met66 BDNF pro-domain alone did not have an effect on cell viability in control cells. (**C**) Cells were treated with Met66 pro-domain of BDNF (200 ng/ml) in combination with 25 nm Aβ_1–42_ for 48–60 h. In the presence of Aβ_1–42_ cell death was increased upon addition of Met66 BDNF pro-domain (*n* = 9) performed. Each bar represents mean ± SEM (*n* = 9). Statistical comparisons were made by paired *t* test. **P < 0.01. (**D**) Twenty-six paired experiments indicated that cell death was higher when a particular preparation of Aβ_1–42_ (25 nm, *n* = 9 preparations of Aβ_1–42_) was supplemented with Met66 BDNF pro-domain (*y*-axis) than for that preparation of Aβ_1–42_ alone (*x*-axis, dashed line indicates the null hypothesis of no effect of the Met66 BDNF pro-domain). (**E**) Similar results were seen when non-differentiated and (**F**) differentiated cells were treated with the Met66 BDNF pro-domain (200 ng/ml) in combination with 1 μm Aβ_1–42_ for 48 h. (**G**) Cells were treated with the Met66 BDNF pro-domain (200 ng/ml) in combination with 25 nm Aβ_1–42_ oligomers for 48–60 h before SDS–PAGE and western blotting with antibodies against p75^NTR^ and sortilin. β-Actin expression was used as a loading control. Western blots indicated that levels of p75^NTR^ but not sortilin were higher following Aβ_1–42_ oligomer treatment in cultures of human neuroblastoma cells. Representative blots are shown (*n* = 3). (**H**) The p75^NTR^ and (**I**) sortilin intensities from the western blots were normalized against the level of β-actin. Each bar represents mean ± SEM (*n* = 3). Statistical comparisons were made by one-way analysis variance test. *P < 0.05.

We now sought to determine whether the synergy between the pro-domain of DNT1 and Aβ, seen in the longevity experiments in *Drosophila*, is also observed in mammalian cell culture. We used oligomeric preparations of Aβ_1–42_ (Supplementary Material, Fig. S3, lanes 2–3) to induce cell death in cultures of SH-SY5Y human neuroblastoma cells and showed that there was a non-linear dose-dependent reduction in cell viability as measured by the MTT assay. Significant cell death (20–30%) was seen 48–60 h after exposure to Aβ_1–42_ oligomer preparation (Fig. 4A). To test whether the BDNF pro-domain could enhance Aβ_1–42_-induced cytotoxicity, we treated cells with both wild type (Val66) and variant (Met66) recombinant pro-domain of BDNF with, or without, 25 nm oligomeric Aβ_1–42_. The MTT results indicated that the variant Met66 BDNF pro-domain alone did not affect cell viability (Fig. 4B); however, the variant BDNF pro-domain (200 ng/ml) combined with Aβ_1–42_ oligomers (25 nm) resulted in greater cell death when compared with Aβ_1–42_ alone (Fig. 4C, *P* < 0.01). When we consider all 26 paired experiments (for *n* = 9 preparations of Aβ), we saw more cell death when a particular preparation of Aβ was supplemented with the variant Met66 BDNF pro-domain when compared with treatment with the same preparation of Aβ_1–42_ alone (Fig. 4D, paired MTT assays of Aβ toxicity ±Met66 BDNF pro-domain). No such interaction was seen between Aβ and the wild-type Val66 pro-domain of BDNF (Supplementary Material, Fig. S4A and B, nine paired experiments, *n* = 3 preparations of Aβ). As expected the mature domain of BDNF alone did protect the cells from Aβ toxicity (Supplementary Material, Fig. S4C and D). These data indicate that, similar to the findings in the fly model, the NT pro-domain acts synergistically with Aβ_1–42_ to exert a toxic effect. Using higher concentrations (1 µM) of Aβ_1–42_, similar results were also seen for non-differentiated (Fig. 4E, nine paired experiments, *n* = 3 preparations of Aβ), differentiated (Fig. 4F, nine paired experiments, *n* = 3 preparations of Aβ) and SH-SY5Y cells. Aβ has previously been reported to increase sortilin and p75^NTR^ levels (24,25) and we sought to confirm this observation in our cultures of SH-SY5Y cells treated with 25 nm oligomeric Aβ_1–42_. Western blots indicated that levels of p75^NTR^, but not sortilin, were higher following Aβ_1–42_ treatment (Fig. 4G–I). The pro-domain of BDNF did not modulate this Aβ_1–42_-mediated upregulation of p75^NTR^ (Fig. 4G and H).

### An elevated ratio of pro- to mature-domain of BDNF correlates with disease status in elderly individuals

**Figure 5.**
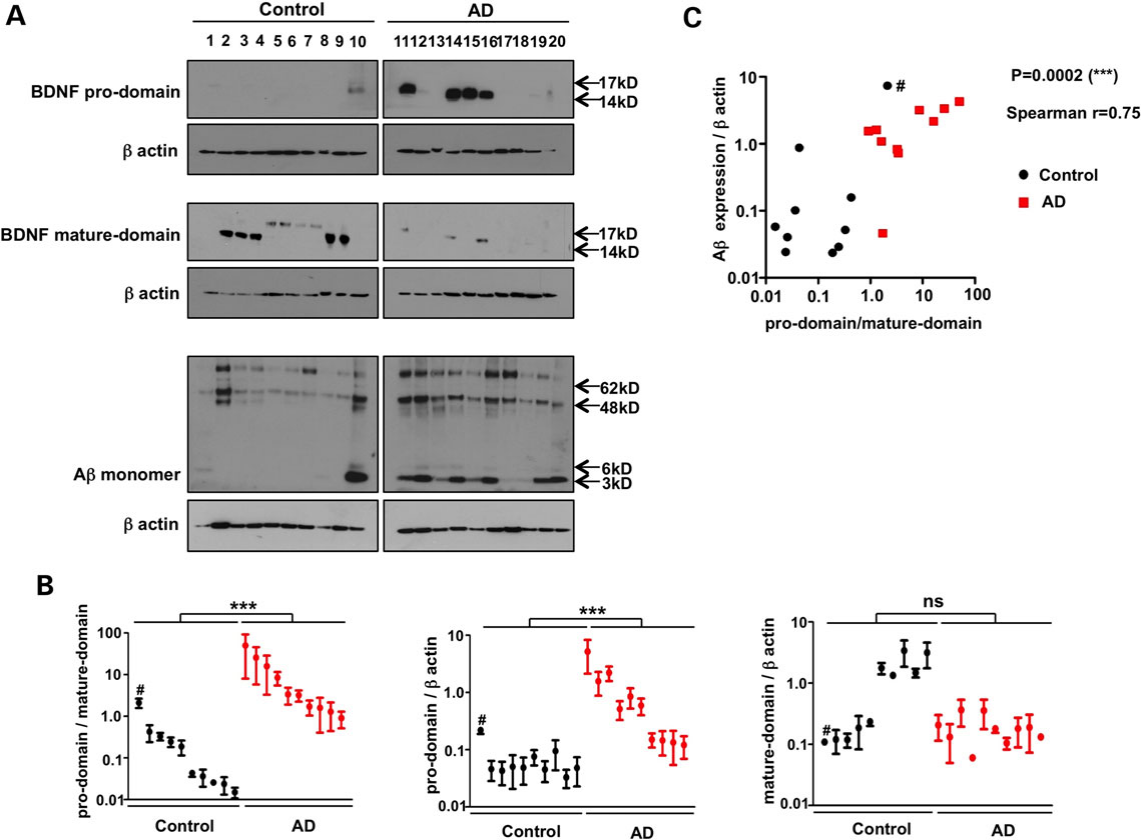
An elevated ratio of pro- to mature-domain BDNF correlates with disease status in elderly individuals. (**A**) Frozen post-mortem hippocampal tissues from 10 patients with advanced AD were homogenized and proteins were separated by SDS–PAGE and then subjected to western blot analysis with antibodies against BDNF pro-domain, mature-domain and Aβ. β-Actin expression was used as a loading control. Western blots show that the levels of BDNF pro-domain were higher, and mature-domain levels lower, in the hippocampal formation of AD cases than in control brains. (**B**) The pro- and mature-domains of BDNF were quantified by densitometry and normalized against the corresponding β-actin band. As expected, mature-domain BDNF levels were, on average, 6-fold lower in cases when compared with controls. In contrast, the levels of pro-domain BDNF were 16-fold higher in cases. Combining these findings as a pro:mature domain ratio yielded a relative difference for cases and controls of 30-fold. (**C**) There was a robust correlation between the BDNF pro:mature domain ratio and the accumulation of urea-solubilizable Aβ in the brain extracts. Of particular note is the control individual marked with #.

We next tested whether the levels of BDNF pro- and mature-domains were different in extracts of age-matched post-mortem hippocampal samples from 10 patients with advanced AD (Braak Stages 5–6) and 10 healthy individuals (Table 1). Western blots of SDS–PAGE gels (Fig. 5A) showed that, as expected, BDNF mature-domain levels were, on average, 6-fold lower in cases when compared with controls (Supplementary Material, Table S1). In contrast, the levels of BDNF pro-domain were, on average, 16-fold higher in cases. Combining these findings as a pro:mature-domain ratio revealed a 30-fold relative difference for cases and controls (Fig. 5B; Supplementary Material, Table S1). Furthermore, we observed a robust correlation between the BDNF pro:mature domain ratio and the accumulation of urea-solubilizable Aβ in the brain extracts (Fig. 5C). Of particular note is the control individual (age 63, Fig. 5B and C marked with #) who, despite being asymptomatic and free of obvious AD pathology, is nevertheless the only control with significant Aβ accumulation, elevated pro-domain levels and a pro:mature domain ratio that overlaps with AD patients.

**Table 1.** Demographics of Alzheimer's disease cases and control individuals (as provided by the source)

Diagnosis	Braak Stage	Age (years)	Gender
1. Controls		89	F
2. Controls		85	M
3. Controls		85	F
4. Controls		89	F
5. Controls		82	F
6. Controls		88	M
7. Controls		67	M
8. Controls		87	F
9. Controls		91	M
10. Controls		63	F
Mean		82.6	
SEM		3.056	
11. AD	5	68	M
12. AD	5	90	M
13. AD	5	84	F
14. AD	5/6	65	F
15. AD	5	84	F
16. AD	5	95	F
17. AD	5	87	M
18. AD	5	92	F
19. AD	5	82	M
20. AD	5	83	F
Mean		83	
SEM		3.059	

Hippocampal tissue was taken from age and sex matched AD cases and controls.

## Discussion

NTs are a major class of molecules promoting neuronal survival in vertebrates. They are synthesized as larger precursor forms that are proteolytically processed to yield a mature, biologically active ligand (6,7). Among the NTs, BDNF has emerged as a major regulator of synaptic plasticity, neuronal survival and differentiation, and also as a potential molecular target for the treatment of neurological disease (26). Several studies indicate that the cortex and hippocampus, areas of the brain associated with learning and memory, not only exhibit extensive amyloid pathology but also show decreased levels of BDNF in AD (14,15,27). Interestingly, precursor and mature forms of BDNF are significantly decreased in preclinical and early stages of AD, and this reduction correlates with clinical neuropsychological scores (15). Low levels of BDNF may favor AD pathogenesis by failing to adequately support neurones and allowing them to succumb to other toxic insults. Furthermore, several studies have linked polymorphisms, specifically Val66Met and Cys270Thr, in the pro-domain of BDNF to an increased risk for AD (28,29).

BDNF exerts many of its neuroprotective effects by binding to the TrkB receptor, a member of the tumour necrosis factor receptor family (9). Indeed, some reports indicate that Aβ may have a negative effect on neuronal survival by down-regulating the TrkB receptor (16). For example, levels of TrkB are reduced in the temporal and frontal cortex of AD brain (30). In addition, BDNF-induced TrkB autophosphorylation and the activation of the downstream enzymes AKT and ERK are all suppressed in the hippocampus of APP/PS1 mice (31).

The consequences of BDNF binding to its alternative receptor p75^NTR^ and sortilin are less well understood, although they are thought to include promotion of myelination (3) and neuronal migration (10) but also neuronal process retraction (11) and neuronal apoptosis (12). It is proposed that the balance between cell death and survival is determined by the relative activity of the precursor versus mature forms of BDNF; indeed, in dorsal root ganglion lesion models in neonatal rats, the signaling appears to be affected by the relative levels of the relevant receptors, namely TrkB, p75^NTR^ and sortilin. Notably, BDNF promotes the death of cultured neurons *in vitro* when p75^NTR^ is upregulated and TrkB downregulated (13). Precursor BDNF can also cause cell death in both *in vitro* and *in vivo* model systems (32) and the prevailing view is that the apoptotic signal is generated by the pro-domain. Similar domain-specific activities have also been observed for NGF; specifically, the precursor form of this related NT preferentially activates p75^NTR^ resulting in apoptosis, while mature NGF preferentially activates TrkA receptor with neurotrophic effects (7).

Mammalian NTs are similar in many ways to their insect orthologues. Sequence analysis has identified *Drosophila* neurotrophin (DNT1), also called Spatzle 2 (Spz 2), as the closest fly orthologue of human BDNF. Like the NTs, the Spz polypeptides are synthesized with a signal peptide, followed by a pro-domain and then the cysteine knot-containing mature domain (33,34). The characteristic NT cysteine knot, formed by antiparallel β-sheets held together by three disulfide bonds, is conserved in the crystal structures of both Spz and NGF (35,36). There is also functional conservation between DNT1, Spz and the mammalian NTs in the nervous system (20,37,38). Indeed, during *Drosophila* embryogenesis DNT1 is expressed in neurones and muscle cells where it promotes neuronal survival and suppresses apoptosis (20). We have shown that the high levels of endogenous DNT1 mRNA that are present during larval development are rapidly suppressed at the beginning of adult life in both control flies and similarly in those expressing various isoforms of the Aβ peptide (Fig. 1).

Because of the functional similarities between mammalian and *Drosophila* NTs, we undertook a study of the interaction between Aβ and DNT1 in the fruit fly. We found that transgenic expression of the DNT1 mature-domain protected flies against Aβ toxicity (Fig. 2) and that this benefit was not due to spurious reductions in Aβ levels (Fig. 3). Similarly, in human SH-SY5Y culture, the mature-domain of BDNF protected cells from Aβ toxicity (Supplementary Material, Fig. S3B). In both the fly and the mammalian models, the pro-domains of both DNT1 and BDNF, while being harmless alone, were nevertheless toxic when added in combination with Aβ (Figs 2 and 4). In SH-SY5Y cells, this synergistic toxic interaction between Aβ and the BDNF pro-domain was only apparent for the Met66 variant and was absent for the wild-type Val66 isoform (Fig. 4; Supplementary Material, Fig. S4A). This finding provides mechanistic underpinning for the clinical observation that the Met66 polymorphism is linked to poor prognosis, particularly in individuals with high Aβ burden on PiB photon emission tomography brain scans (18,39).

The expression of the receptor p75^NTR^ is upregulated in both SH-SY5Y cells following Aβ treatment and also in transgenic mice expressing the human APPswe transgene (Fig. 4G and H) (40,41). Aβ treatment also enhances sortilin expression via p75^NTR^, which is thought to activate the downstream effectors JNK and Rho (24,25), resulting in apoptotic cell death. Furthermore, the accumulation of Aβ in humans is also accompanied by an increased hippocampal membrane-associated p75^NTR^ (42). However, it seems unlikely that Aβ binds directly to p75^NTR^ (43), rather we suggest that Aβ amplifies the pro-apoptotic signaling of pro-domain BDNF by upregulating p75^NTR^ in the human SH-SY5Y cells (Fig. 4G and H).

Our subsequent work with post-mortem hippocampal tissue from age-matched healthy elderly and patients with AD has underlined the clinical significance of these experimental findings. As expected, the mature-domain of BDNF is reduced in AD when compared with control brain tissue (15). In our series of 10 cases and controls, we found that control subjects fell into two groups—those with levels of BDNF mature-domain that are up to 20 times higher than AD cases (Fig. 5); however, an equal number of controls have mature-domain levels that are equivalent to AD patients. The separation between cases and controls is better when we measure BDNF pro-domain levels: cases have elevated levels of pro-domain, spread over almost 2 orders of magnitude while all but one of the controls (outlier labelled with #) are closely grouped. The average pro-domain level is ∼16-fold higher in AD cases when compared with controls. These reciprocal changes in the levels of pro- and mature-BDNF are surprising considering that the peptides are generated stoichiometrically. Receptor-mediated clearance is an unlikely explanation because the levels of TrkB are low and p75 high in AD cases when compared with controls (30,31,42). Conceivably, in AD the BDNF pro-domain is being stabilized by binding to another protein (44); this intriguing possibility requires further investigation.

When these relative changes in pro- and mature-domains are combined as a ratio, we see that cases and controls have an average 30-fold difference and only one of the controls overlaps with the AD range. This particular control individual is interesting because, despite being symptom-free and relatively young (aged 63), she was the only control to have accumulated significant Aβ. In fact, she had the highest level of Aβ amongst all cases and controls. That this control individual's pro:mature domain ratio was in the AD range raises the tantalizing possibility that we are able to predict disease changes before the onset of symptoms.

In conclusion, we have shown for the first time that the pro- and mature-domains of NTs have opposing effects on Aβ neurotoxicity *in vitro* and *in vivo*. The mature-domains of NTs protected against Aβ_1–42_ toxicity, whereas the pro-domains acquired a toxic role in the presence of Aβ_1–42_. In our study of post-mortem clinical brain samples, the ratio of pro- to mature-domain of BDNF was significantly higher in patients with AD when compared with controls. Taken together, the finding that patients with AD have elevated levels of BDNF pro-domain underlines the importance of its synergistic toxic interaction with Aβ. Conceivably, individuals with an unfavourable pro:mature domain ratio could be targeted with therapy aimed at restoring a more neurotrophic environment in the brain.

## Materials and Methods

### Fly stocks and maintenance

All fly stocks were maintained at 25 or 29°C on a 12:12-h light:dark cycle at constant 60% humidity on a standard cornmeal-based *Drosophila* medium. Adult-onset neuronal-specific expression of DNT1, human Aβ_1–40_, Aβ_1–42_ and Aβ_1–42_ with Arctic mutation (Arc Aβ_1–42_) was achieved by using the gal4-UAS system. The elav^C155^-Gal4 driver was used for all experiments. The transgenic fly lines carrying human Aβ peptides have been described previously (45). The transgenic fly lines carrying three forms of DNT1: (1) full-length DNT1 (UAS-DNT1 full length); (2) pro-domain of DNT1 (lacking the Cysknot) (UAS-DNT1 pro-domain); (3) Cysknot DNT1 (UAS-DNT1 mature-domain, lacking the C-terminal extension) as described by Zhu *et al*. (20) were kind gifts from Dr Alicia Hidalgo (University of Birmingham, UK). Flies transgenic for anti-DNT1 hairpin loop RNAi constructs were obtained from the Vienna Drosophila Resource Center (Transformant ID: 26115, Construct ID: 10831).

### Quantitative RT-PCR

Total RNA was extracted from 50 fly heads using the RNA isolation kit (Quiagen) according to the manufacturer's instructions. The concentration of total RNA purified for each sample was measured using a spectrometry on the basis of optical density measurements. One microgram of total RNA was then subjected to DNA digestion using DNAse I (Invitrogen), immediately followed by reverse transcription using the Superscript II system (Promega) with oligo(dT) primers. Quantitative PCR was performed using an SYBR Green PCR mixture (Bio-Rad) according to the manufacturer's instructions. Each sample was analysed in triplicate with both target gene (DNT1) and control gene (RP49) primers in parallel. The DNT1 primers amplified the cDNA coding for the Cysknot: forward 5′-TAGACGACTGCAATTAAAAACAA-3′; reverse 5′-GCCTGTTCAAACTTTAATTCGT-3′. The RP49 primers were as follows: forward 5′-ATGACCATCCGCCCAGCATCAGG-3′; reverse 5′-ATCTCGCCGCAGTAAACG-3′.

### Longevity assay

Male flies carrying UAS-Arc Aβ_1–42_ (Aβ_1–42_ with E22G substitution), UAS-DNT1 (various forms of the DNT1 protein), UAS-DNT1RNAi (RNAi against DNT1) or DNT1^null^ (null mutant) were crossed with virgin elav^C155^-Gal4 driver lines. From the progeny, mated female flies were collected 24 h post-eclosion and divided between 10 tubes, each containing 10 flies. The flies were then cultured with the surviving flies counted on Days 1, 3 and 5 of a 7 days cycle. The median survival of the flies in each of the 10 tubes was plotted for each treatment condition. The mean was indicated ±SEM. The statistical significance of differences in longevity was determined by considering each tube to be an independent estimate of the population median survival and using one-way analysis variance test (*n* = 10).

### Locomotor activity assay

Flies carrying UAS-Arc Aβ_1–42_, UAS-DNT1, UAS-DNT1RNAi or DNT1^null^ were crossed with elav^C155^-Gal4 driver lines. From the progeny, mated female flies were collected 24 h post-eclosion and for each condition five flies were placed in each of three different tubes of 2 cm diameter and 10 cm height. Three 30 s videos were taken on Days 1, 3, 7, 9, 12, 14 and 16 using the iFly apparatus and the walking velocity of the flies’ negative geotactic response was determined as described previously (46). Experiments were repeated more than twice, and representative results are shown.

### Generation of Aβ oligomers

β-Amyloid (1–42) prepared in HFIP was purchased from Eurogentec. Oligomeric amyloid was prepared as described by Dahlgren *et al*. (47). Briefly, Aβ that was lyophilized as a hexafluoroisopropanol film was dissolved in dry dimethyl sulfoxide (DMSO, Sigma) to a concentration of 1 mm and then sonicated for 180 s. Dry DMSO was added to a concentration of 100 or 25 μm and Ham's F-12 (Invitrogen) was then added to bring the peptide to a final concentration of 10 or 2.5 μm, and the samples were rotated on a rotary shaker at 4°C for 5 days and then RT for 2 days.

### Cell culture and treatment

The human neuroblastoma SH-SY5Y cell line from European collection (ECACC) were cultured in medium containing equal volumes of minimum essential medium (MEM, Gibco) and Nutrient Mixture Ham's F-12 (Gibco), supplemented with 1% (v/v) penicillin/streptomycin (antibiotics, Invitrogen), 1% (v/v) non-essential amino acids (Gibco) and 10% (v/v) foetal bovine serum (FBS, Gibco) in a humidified atmosphere containing 5% (v/v) CO_2_ at 37°C. Cells were plated 3 × 10^4^ cells/cm^2^ into culture dishes and maintained in growing medium. Twenty four hours after plating, cells were washed and maintained in 0.5% (v/v) FBS-containing medium for 18 h. After that cells were replaced with 0.2% (v/v) FBS-containing medium and then incubated further 48–72 h in the presence of Aβ_1–42_ oligomers with or without recombinant human wild type (Val66) or variant (Met66) BDNF pro-domain (Alomone labs). For differentiation of SH-SY5Y cells, cell were plated 3.5 × 10^4^ cells/cm^2^ into culture dishes and maintained in growing medium. Twenty four hours after plating, cells were washed and replaced with MEM, 1% (v/v) N-2 supplements (Invitrogen), 2 mm l-glutamine (Invitrogen), 1% (v/v) antibiotics and 10 μm all-*trans* RA (Sigma). The medium was replaced every 2–3 days. After 5–6 days of RA differentiation, fresh medium was provided and incubation continued for a further 48 h in the presence of Aβ_1–42_ oligomers with or without recombinant human variant Met66 BDNF pro-domain.

### Measurement of *in vitro* cell viability

Cell viability was measured using the 3-(4,5-dimethylthiazol-2-yl)-2,5-diphenyltetrazolium bromide (MTT)-based cytotoxicity assay (Promega). Cells were seeded in 24-well plates and then treated with increasing dose of Aβ_1–42_ oligomers or recombinant BDNF, or both reagents at adequate concentrations and then maintained for 60–72 h in a 5% (v/v) CO_2_ incubator. MTT was added to all wells and allowed to incubate at 37°C for 4 h, followed by cell lysis and spectrophotometric measurement at 570 nm.

### Cell and tissue extraction

The cells were lysed in cell lysis buffer (Abcam) containing 0.216% (w/v) β-glycerophosphate, 1 mm sodium orthovanadate, 1 μg/ml leupeptin, 1 mm EGTA, 10% (v/v) Triton-X-100, 50 mm Tris–HCl, pH 7.5, 150 mm NaCl, 1 mm sodium EDTA, 1.12% (w/v) sodium pyrophosphate decahydrate and supplemented with phenylmethylsulfonyl fluoride (Sigma), homogenized, sonicated and centrifuged at 20 000*g* for 30 min at 4°C. The supernatant was then retained. Frozen hippocampal tissue (2–3 mg) was homogenized in ice-cold RIPA buffer containing Tris–HCl buffer, pH 7.5, 105 mm NaCl, 1% (v/v) NP-40, 1% (v/v) Triton X-100, 1% (w/v) sodium deoxycholate, 0.1% (w/v) SDS, 2 mm EDTA supplemented with protease inhibitor cocktail (Roche), sonicated for 480 s. The tissue homogenates were centrifuged at 18 000*g* for 20 min at 4°C and the supernatants were centrifuged again at 18 000*g* for 10 min at 4°C. The protein concentration was determined using the DC Protein Assay Kit (Bio-Rad).

### SDS–PAGE and western blots

Samples for western blotting were diluted into the 4× NuPage LDS sample buffer (Invitrogen). For western blots of Aβ from human brain samples the buffer was supplemented with 8 m urea. The samples were boiled and equal amounts of protein were loaded on to each well of NuPAGE 4–12% (w/v) Bis–Tris Gel and transferred to a 0.45 μm nitrocellulose membrane. The membranes were fixed with 2.5% (v/v) glutaraldehyde (Sigma) in PBS, pH 7.4, for detecting the BDNF pro-domain. The membranes were blocked with 5% (w/v) milk, incubated with primary antibodies against Aβ (6E10; Signet Laboratories), p75^NTR^ (Millipore), Sortilin (Abcam), BDNF pro-domain (GeneCopoeia), BDNF mature-domain (Santa Cruz Biotechnology) or β-actin (Abcam). The membranes were then washed and then incubated with appropriate secondary antibody and developed using ECL detection reagents (Thermo Scientific) before exposing to film (Sigma-Aldrich). Film was digitized and bands were quantified in Image J by measuring the mean grey value. For each western blot, one representative of at least three independent experiments was shown.

### Statistical analysis

All data are expressed as means ± SD or means ± SEM from at least three independent experiments. For comparison of multiple groups, the one- or two-way analysis of variance (ANOVA) with a Tukey's *post hoc* test for was used to define statistically significant differences among the groups. The differences between two samples were detected with the two-tailed Mann–Whitney test or Student *t*-test. Probability values <0.05 were considered as significant.

## Supplementary Material

Supplementary Material is available at *HMG* online.

## Funding

This work was supported by the Korea-UK Alzheimer's disease research consortium program from the Ministry of Health and Welfare, Republic of Korea (J.Y.L.) and the Wellcome Trust (082604/2/07/Z; D.C.C.) and an Alzheimer's Research UK Senior Research Fellowship (ART-SRF2010-2; D.C.C.). Funding to pay the Open Access publication charges for this article was provided by MedImmune Ltd.

## Supplementary Material

Supplementary Data
