# Supplementary material for: The pro-domains of neurotrophins, including BDNF, are linked to Alzheimer's disease through a toxic synergy with Aβ
Source: Hum Mol Genet. 2015 May 7;24(14):3929–38. doi: 10.1093/hmg/ddv130 (PMC4476443; doi:10.1093/hmg/ddv130)
Supplement: Supplementary Data [file supp_24_14_3929__index.html]

The pro-domains of neurotrophins, including BDNF, are linked to Alzheimer's disease through a toxic synergy with Aβ — The pro-domains of neurotrophins, including BDNF, are linked to Alzheimer's disease through a toxic synergy with Aβ — Supplementary Data 

# The pro-domains of neurotrophins, including BDNF, are linked to Alzheimer's disease through a toxic synergy with Aβ

## Supplementary Data

Supplementary Data

- Supplementary Data - Docx file
- Supplementary Figure 1 - png file
- Supplementary Figure 2 - png file
- Supplementary Figure 3 - png file
- Supplementary Figure 4 - png file
- Supplementary Table 1 - png file
