# Supplementary material for: The pro-domains of neurotrophins, including BDNF, are linked to Alzheimer's disease through a toxic synergy with Aβ
Source: Hum Mol Genet. 2015 May 7;24(14):3929–38. doi: 10.1093/hmg/ddv130 (PMC4476443; doi:10.1093/hmg/ddv130)
Supplement: Supplementary Data [file supp_ddv130_ddv130supp.docx]

**The pro-domains of neurotrophins, including BDNF, are linked to Alzheimer’s disease through a toxic synergy with Aβ**

Jung Yeon Lim^1^, Charles P. Reighard^2^, Damian C. Crowther^1,3*^

^1^University of Cambridge, Department of Genetics, Downing Street, Cambridge, CB2 3EH, UK

^2^University of Cambridge, Department of Biochemistry, Tennis Court Road, Cambridge, CB2 1GA, UK

^3^MedImmune Limited, Aaron Klug Building, Granta Park, Cambridge, CB21 6GH, UK

*To whom correspondence should be addressed.

Tel: +44 1223 760346 ; Fax: +44 1223 654400

E-mail: dcc26@cam.gen.ac.uk

**Fig. S1. DNT1 deficiency has little impact on functional phenotypes in adult flies.**

(**A**) DNT1 and RP49 mRNA were measured at days 0 (D0) and 10 (D10) by quantitative real-time PCR in control flies and those in which DNT1 was knocked down using DNT1-RNAi or by introducing a single DNT1^null^ allele into the genetic background (DNT1^-^/^+^). Both approaches to DNT1 knockdown resulted in lower levels of endogenous DNT1 mRNA during adult life. Data are expressed as the ratio of DNT1 to RP49 mRNA levels (+/- SD). Statistical comparisons were made by one-way analysis variance test. *** *p* < 0.001. (**B**) Median survival times (+/- SEM) of control flies and those expressing either DNT1-RNAi or DNT1^-^/^+^. The flies were cultured at 25°C. Flies with DNT1 knockdown had a normal life span as compared to wild type flies. (**C**) Median survival time (+/- SEM) of control flies, Arc Aβ_1-42_ flies, and Arc Aβ_1-42_ flies expressing either DNT1-RNAi or DNT1^-^/^+^. The flies were cultured at 25°C. DNT1 knockdown did not affect the longevity of Arc Aβ_1-42_ flies. Statistical comparisons were made by one-way analysis variance test. *** *p* < 0.001.

(**D**) The mean climbing velocity of control flies was not affected by DNT1-RNAi or DNT1^-/+^. Mean climbing velocity +/- SEM. (**E**) The mean climbing velocity (+/- SEM) of Arc Aβ_1-42_ flies was significantly reduced as compared to controls (circles vs. squares). Statistical comparisons were made by two-way analysis variance test. ** *p* < 0.01 and *** *p* < 0.001. Neither DNT1-RNAi (triangles) nor DNT1^-^/^+^ (inverted triangles) modifies this effect.

**Fig. S2. The subdomains of DNT1 have differential effects on neurotoxic phenotypes in the fly.**

(**A**) Expression of full-length and mature-domain of DNT1 prolonged the lifespan of control flies, whereas DNT1 pro-domain had no effect. (**B**) The over-expression of DNT1 mature-domain significantly increased median survival of Arc Aβ_1-42_ flies while DNT1 pro-domain further reduced median survival by 30%. For both panels the flies were cultured at 25°C. Each graph presents the mean of ten estimates of median survival +/- SEM. Statistical comparisons were made by one-way analysis variance test. **, *p* < 0.01 and ***, *p* < 0.001.

**Fig. S3. Monomeric and oligomeric Aβ_1-42_ conformers visualized by western blotting.**

Aβ_1-42_ oligomers were prepared in DMSO and F-12 and then incubated at 4°C for 5 d and then RT for 2 d. 0.2 μg of Aβ_1-42_ monomer (lane 1), 0.1, and 0.2 μg of Aβ_1-42_ oligomers (lanes 2 and 3) were subjected to western blotting and visualized with anti-Aβ antibody (6E10). A ladder of Aβ42 immunoreactive bands were observed in Aβ_1-42_ oligomer lanes but not in the monomer preparation.

**Fig. S4. Wild-type (Val66) BDNF pro-domain does not interact with Aβ_1-42_ but BDNF mature-domain protects cells against Aβ_1-42_ toxicity in SH-SY5Y human neuroblastoma cell cultures.**

(**A**) Cells were treated with wild type (Val66) recombinant pro-domain of BDNF (200 ng/ml) in combination with 25 nM Aβ_1-42_ for 48−60 h. The effect of BDNF pro-domain on Aβ_1-42_ toxicity was analyzed for all MTT experiments (*n* = 3) performed. (**B**) No such interaction was seen between Aβ and the wild-type (Val66) BDNF pro-domain (9 paired experiments, *n* = 3 preparations of Aβ). (**C**) Cells were treated with recombinant mature-domain of BDNF (200 ng/ml) in combination with 25 nM Aβ_1-42_ for 48−60 h. The effect of BDNF mature-domain on Aβ_1-42_ toxicity was analyzed for all MTT experiments performed. Each bar represents mean ± SEM (*n* = 4). Statistical comparisons were made by paired *t* test. ** *p* < 0.01. (**D**) Ten paired experiments indicated that cell death was lower when a particular preparation of Aβ_1-42_ (25 nM, *n* = 4 preparations of Aβ_1-42_) was supplemented with BDNF mature-domain (y-axis) than for that preparation of Aβ_1-42_ alone (x-axis, dashed line indicates the null hypothesis of no effect for the BDNF mature domain).

**Table S1. Levels of Aβ and the pro- and mature-domains of BDNF in cases of Alzheimer's disease and control individuals.**

Aβ and BDNF subdomains were quantified by densitometry and normalized against the corresponding β-actin band in Alzheimer’s disease cases and controls.
