# Supplementary figures and images for: The pro-domains of neurotrophins, including BDNF, are linked to Alzheimer's disease through a toxic synergy with Aβ
Source: Hum Mol Genet. 2015 May 7;24(14):3929–38. doi: 10.1093/hmg/ddv130 (PMC4476443; doi:10.1093/hmg/ddv130)

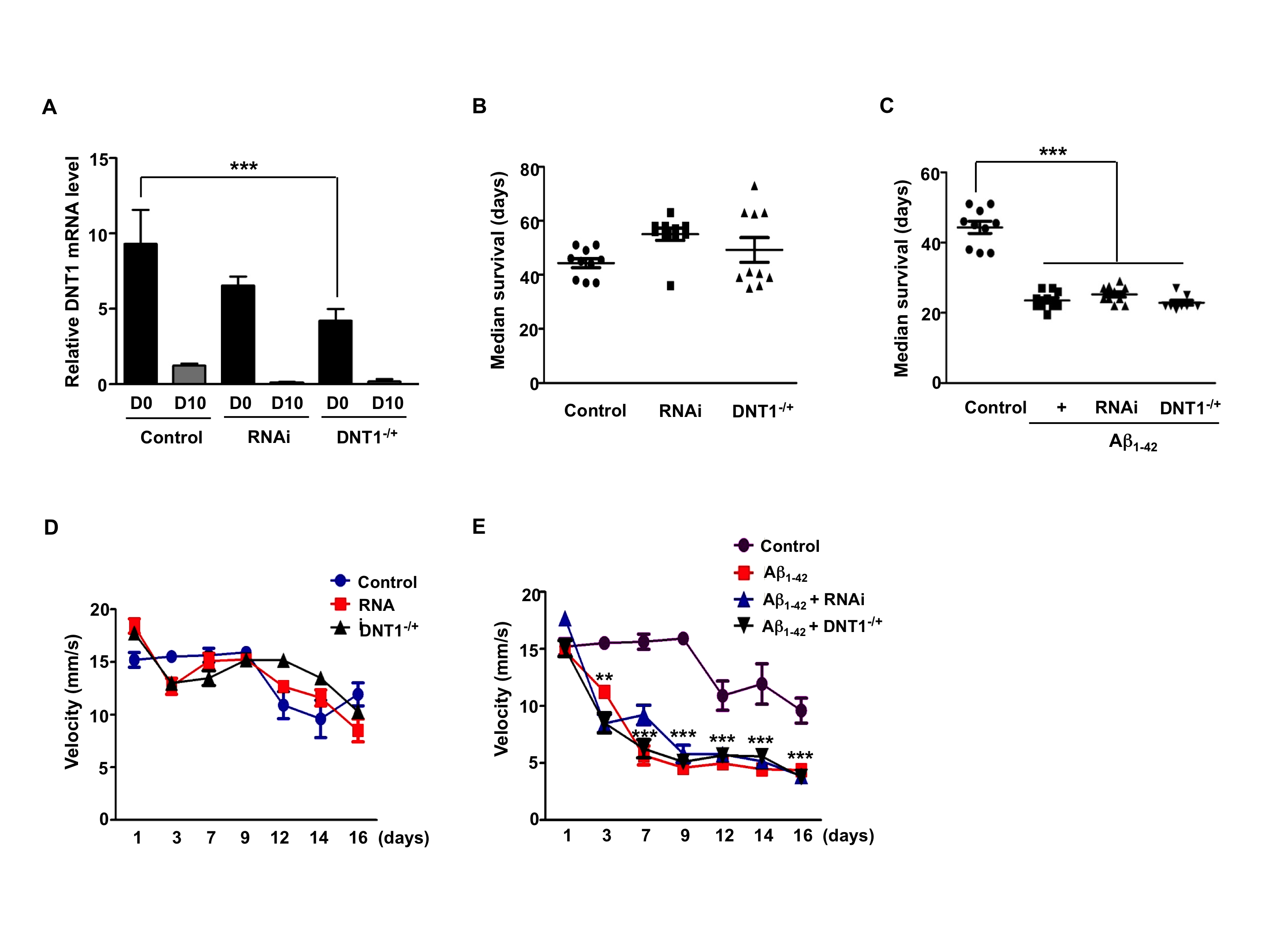

Supplement: Supplementary Data [file supp_ddv130_ddv130supp_fig1.png]

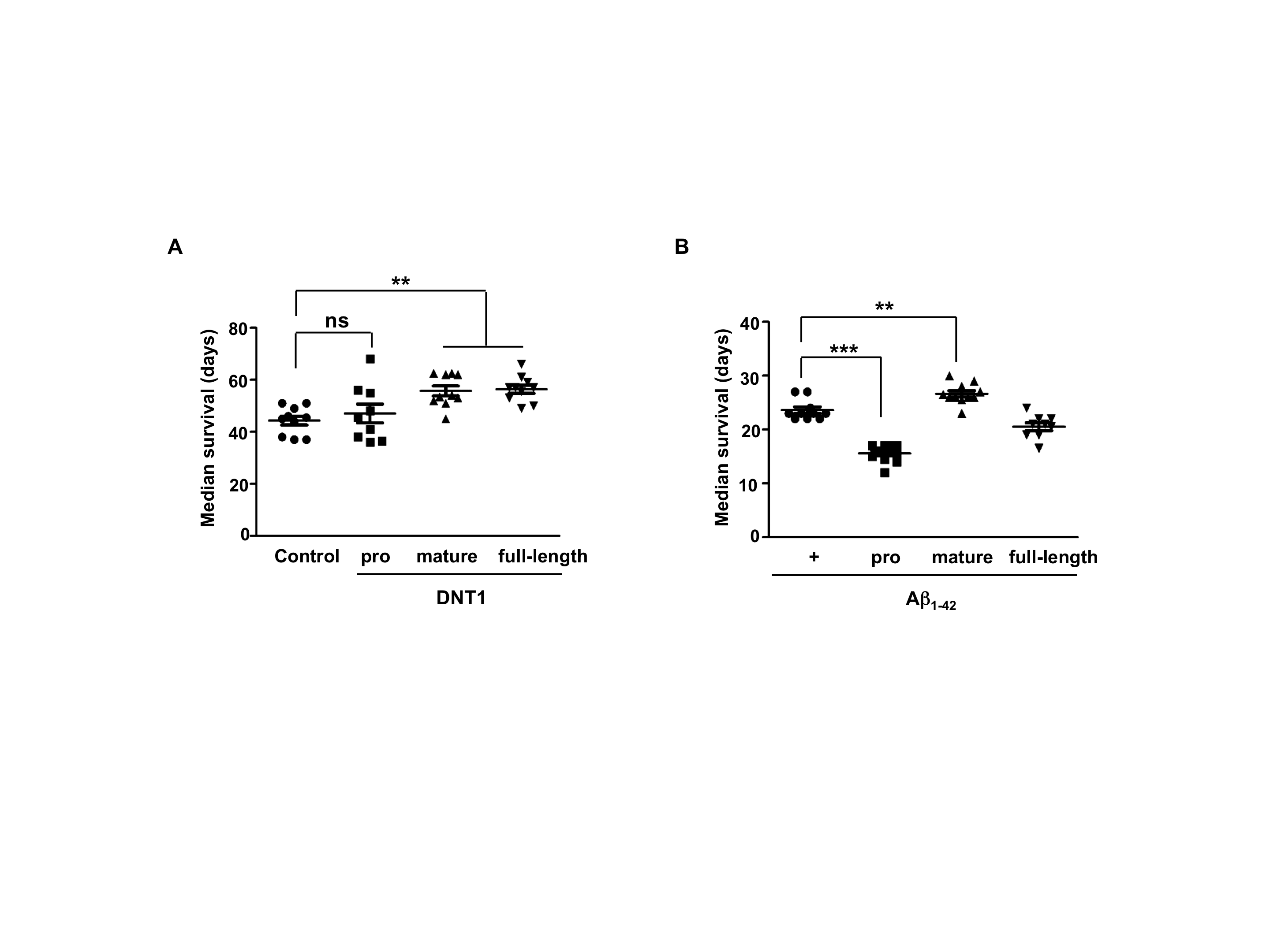

Supplement: Supplementary Data [file supp_ddv130_ddv130supp_fig2.png]

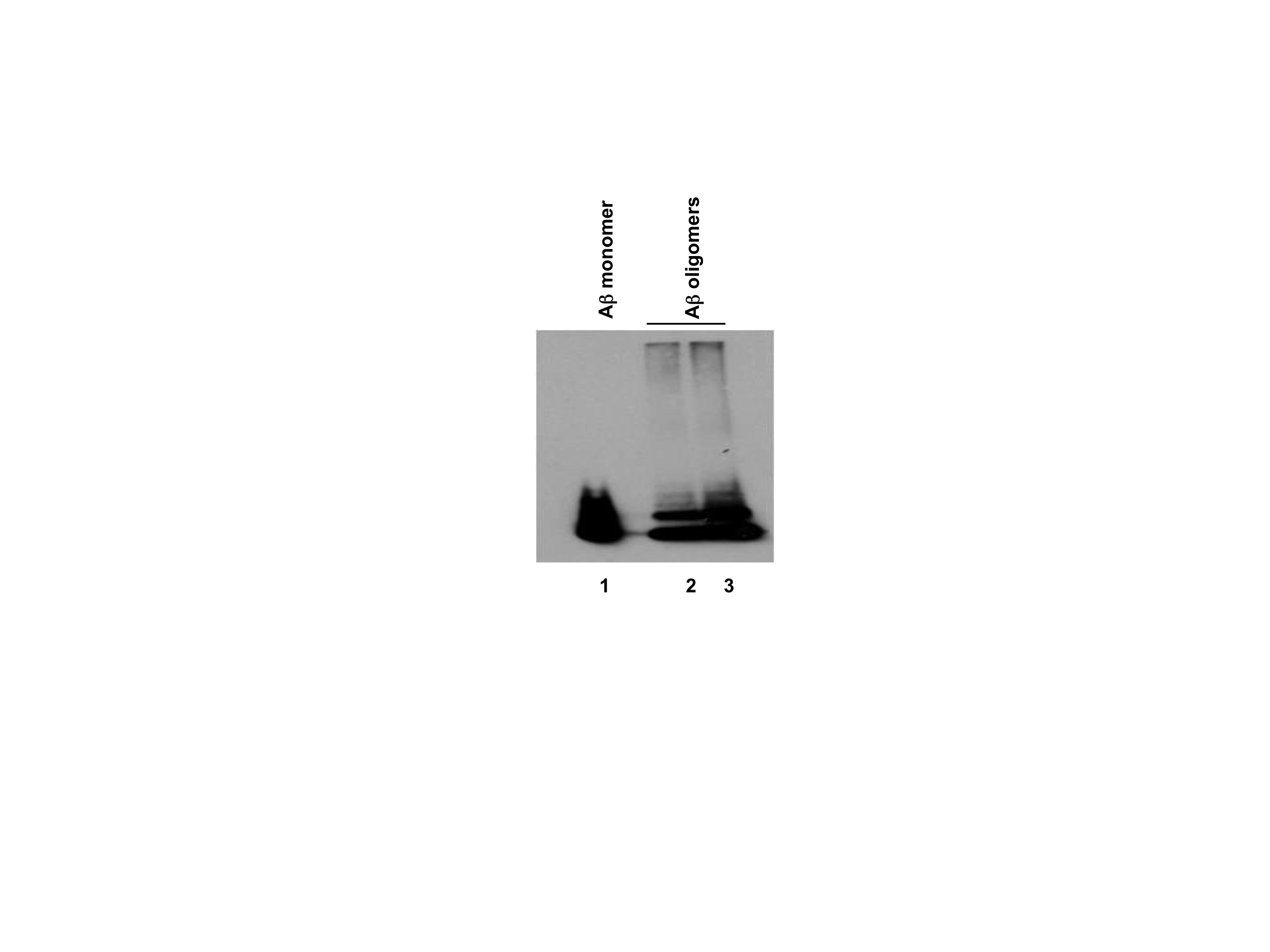

Supplement: Supplementary Data [file supp_ddv130_ddv130supp_fig3.png]

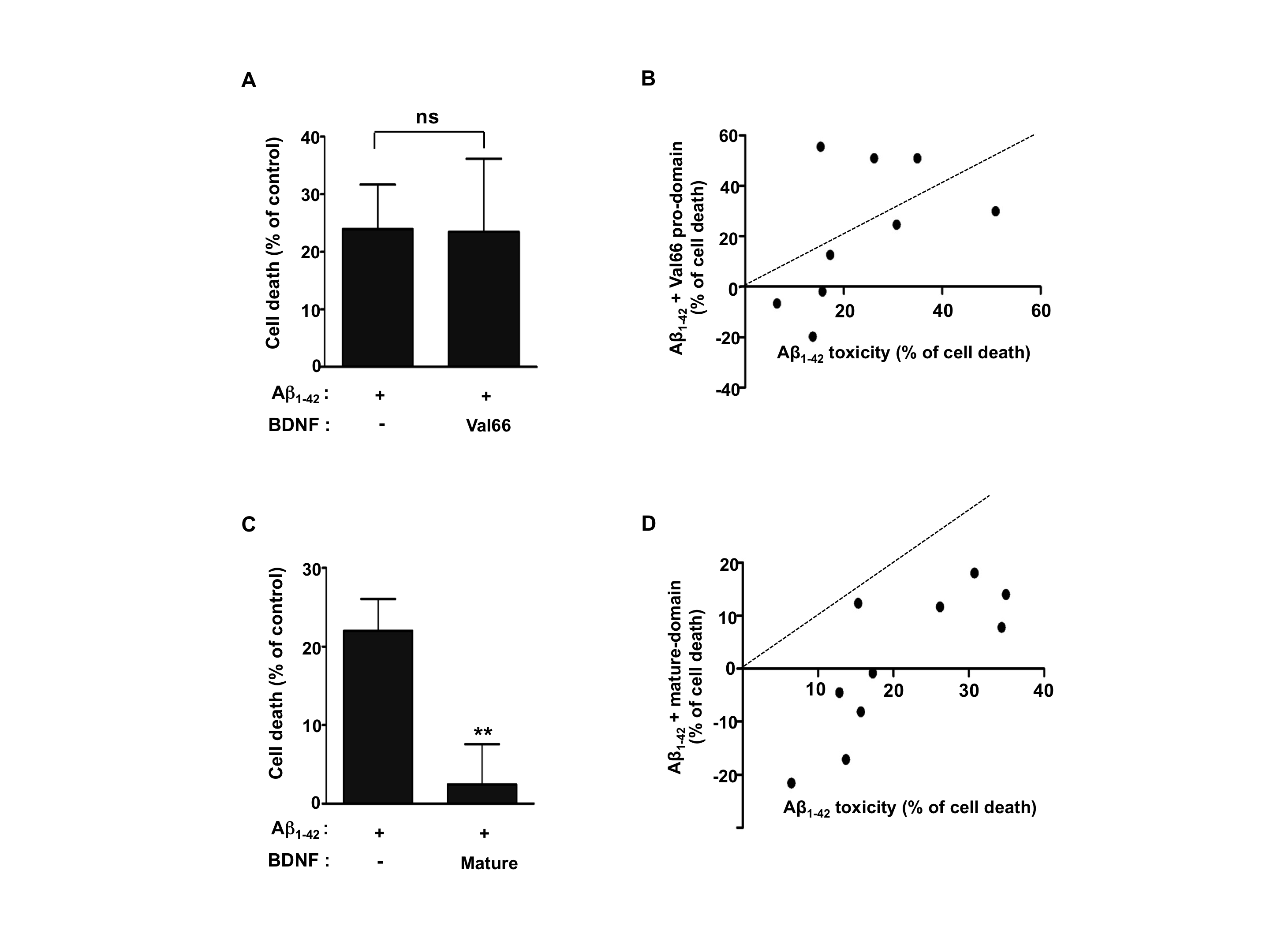

Supplement: Supplementary Data [file supp_ddv130_ddv130supp_fig4.png]

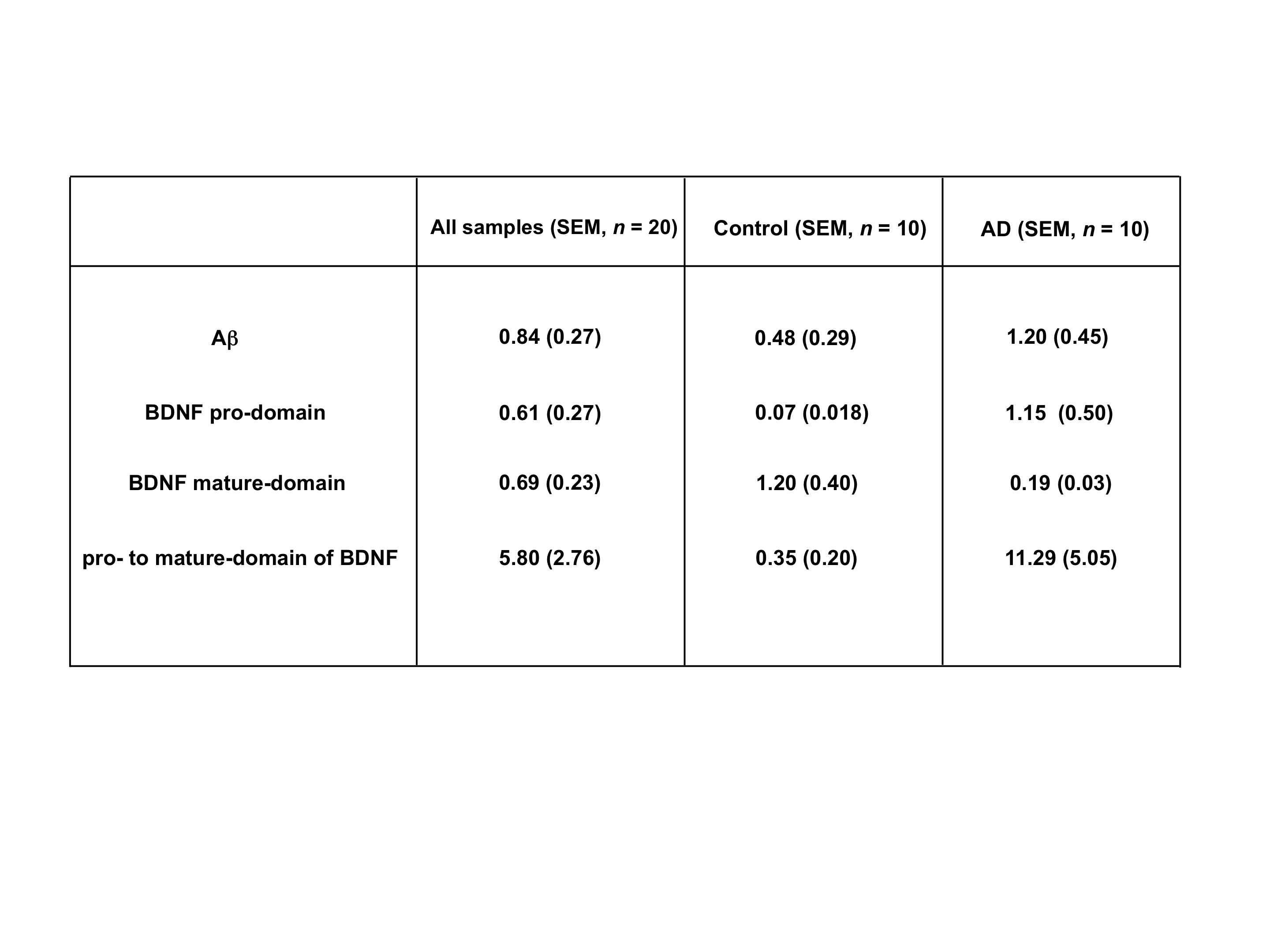

Supplement: Supplementary Data [file supp_ddv130_ddv130supp_table1.png]
